# External Validation of the Oakland Score for Acute Lower Gastrointestinal Bleeding

**DOI:** 10.7759/cureus.57264

**Published:** 2024-03-30

**Authors:** Ismail Raqi, Pascal Potier, Jean-Paul Lagasse

**Affiliations:** 1 Hepatology and Gastroenterology Department, Ibn Rochd University Hospital, Casablanca, MAR; 2 Hepatology and Gastroenterology Department, University Hospital of Orleans, Orleans, FRA

**Keywords:** emergency department, external validation, safe discharge, risk stratification, oakland score, gastrointestinal bleeding

## Abstract

Introduction

Acute lower gastrointestinal bleeding (LGIB) presents challenges in emergency settings, with incidence influenced by demographic shifts and anticoagulant usage. The Oakland score aids in risk stratification for safe discharge based on clinical and laboratory parameters. However, external validation remains limited.

Methods

This study validated the Oakland score in a French cohort of patients with acute LGIB and assessed the discriminatory value of the score using the area under the curve (AUC) and then its sensitivity and specificity.

Results

A retrospective examination of 343 patient records that satisfied the inclusion criteria showed a median score of 14 points and good discriminatory capacity (area under the receiver operating characteristic (AUROC) curve: 0.83). There was low sensitivity (20.9%) for safe discharge but good specificity (98.5%) when using an 8-point threshold. With a 9-point threshold, the sensitivity was increased to 36.5%, while the specificity remained at 95%.

Conclusion

Identifying low-risk LGIB patients is accomplished without sacrificing sensitivity by increasing the Oakland score threshold to 9 points. This modification improves patient safety and resource allocation in the emergency room and has been verified by other large series. For wider implementation, additional validation and long-term outcome evaluations are required.

## Introduction

Consultations for acute lower gastrointestinal bleeding (LGIB) are common, especially in emergency rooms. According to estimates, the incidence ranges from 33% to 87% per 100,000 inhabitants/year [1-2], with an upward trend due to population aging and a rising number of anticoagulant prescriptions. However, not all LGIB patients need urgent treatments including surgery, mesenteric embolization, therapeutic endoscopy, or blood transfusion. In fact, LGIB typically takes a quit course. In 80-85% of instances, the bleeding ceases spontaneously [3-4], while the death rate ranges from 2% to 4% [5-6].

Identifying patients with minimal risk of harm who are suitable for outpatient examination is therefore an important objective.

According to the European Society of Gastrointestinal Endoscopy (ESGE) and the British Society of Gastroenterology, a clinician may choose to discharge a patient for outpatient investigation based on an Oakland score of ⩽8 points if the patient presents with a self-limited hemorrhage and no adverse clinical features [7-8]. Using a nationally representative sample of UK patients, the Oakland score was developed in 2017 to identify patients who have a low risk of negative outcomes and can, thus, be safely discharged to avoid hospitalization [9]. For risk stratification, this scoring system uses laboratory and clinical information.

It has seven factors that range from 0 to 35 points: age, gender, previous hospitalization for LGIB, findings of the digital rectal examination, heart rate, systolic blood pressure, and hemoglobin concentration. It is 0-35 points in range. An increased score signifies a greater likelihood of an adverse outcome.

The Oakland score has received only a limited amount of external validation [9-10], although the ESGE has recommended employing the tool for assessing patients with acute LGIB [7]. Therefore, the objective of this study was to determine the sensitivity and specificity of the threshold established by the derivation study and to study the other thresholds while externally validating the Oakland score in a cohort of French patients with acute LGIB.

## Materials and methods

Setting and study design

To identify patients with acute LGIB, records from the University Hospital of Orleans in Orleans, France, were consulted. The study was approved by the Research Review Council of the hospital. According to the French legal and ethical guidelines for research, an information notice and a non-objection notice were sent to each participant. The study was conducted following the guidelines outlined in the Strengthening the Reporting of Observational Studies in Epidemiology (STROBE) statement [11].

Study population and data collection

The International Classification of Diseases, Tenth Revision (ICD-10) code (K62.5) that was consistent with a hematochezia was used to retrospectively identify adult patients (aged ≥16 years) who were admitted to the hospital with acute LGIB between January 2, 2020, and August 30, 2023. To remain within the setting of an acute hemorrhage, only cases of hematochezia lasting under three days were considered. Acute LGIB should be the main reason for consultation.

Patients with severe bleeding defined by upper gastrointestinal hemorrhage justifying an urgent endoscopic hemostasis procedure or a shock index >1 were excluded. Patients whose records lacked sufficient information to calculate their scores were also excluded. For each patient, information was collected on their demographics, vital signs, blood test results, therapies, and outcomes. Heart rate (measured in beats per minute), systolic blood pressure (measured in mmHg), and hemoglobin concentration (measured in g/L) were extracted from the results of each patient's first recorded set of vital signs and blood tests.

Outcomes

The absence of all of the following adverse events was considered safe discharge: rebleeding defined as bleeding in the first four hours of evaluation, red blood cell transfusion, therapeutic intervention to control bleeding, defined as endoscopic, radiological, or surgical hemostasis, in-hospital death from any cause, and readmission with LGIB within 28 days.

After determining the discriminatory capacity of the score in our population by examining the receiver operating characteristic (ROC) curve, the major endpoint is the identification and analysis of the sensitivity and specificity of the threshold of 8, followed by an evaluation of a different threshold in light of the findings.

Sample size calculation

Since the area under the curve (AUC) is the main outcome metric, a 95% confidence interval (CI) with a width of ≤0.1 can only be obtained with a sample size of at least 265. This conclusion is supported by the results from Oakland et al.'s validation trial [9], which showed an AUC of 0.87 and a percentage of individuals who were discharged safely of 0.479. Furthermore, the formulas suggested by Zhou et al. [12] serve as a guide for the calculations.

Statistical analysis

Sensitivity and specificity estimates, along with 95% CI, were calculated for various decision thresholds of the Oakland score. The performance evaluation of the Oakland score was conducted using the AUC. An optimal cutoff score, ensuring at least 95% specificity for safe discharge of low-risk LGIB patients while maximizing sensitivity, was determined. Statistical analysis was conducted using IBM SPSS Statistics for Windows, Version 26.0 (Released 2019; IBM Corp., Armonk, New York, United States).

## Results

**Figure 1:**
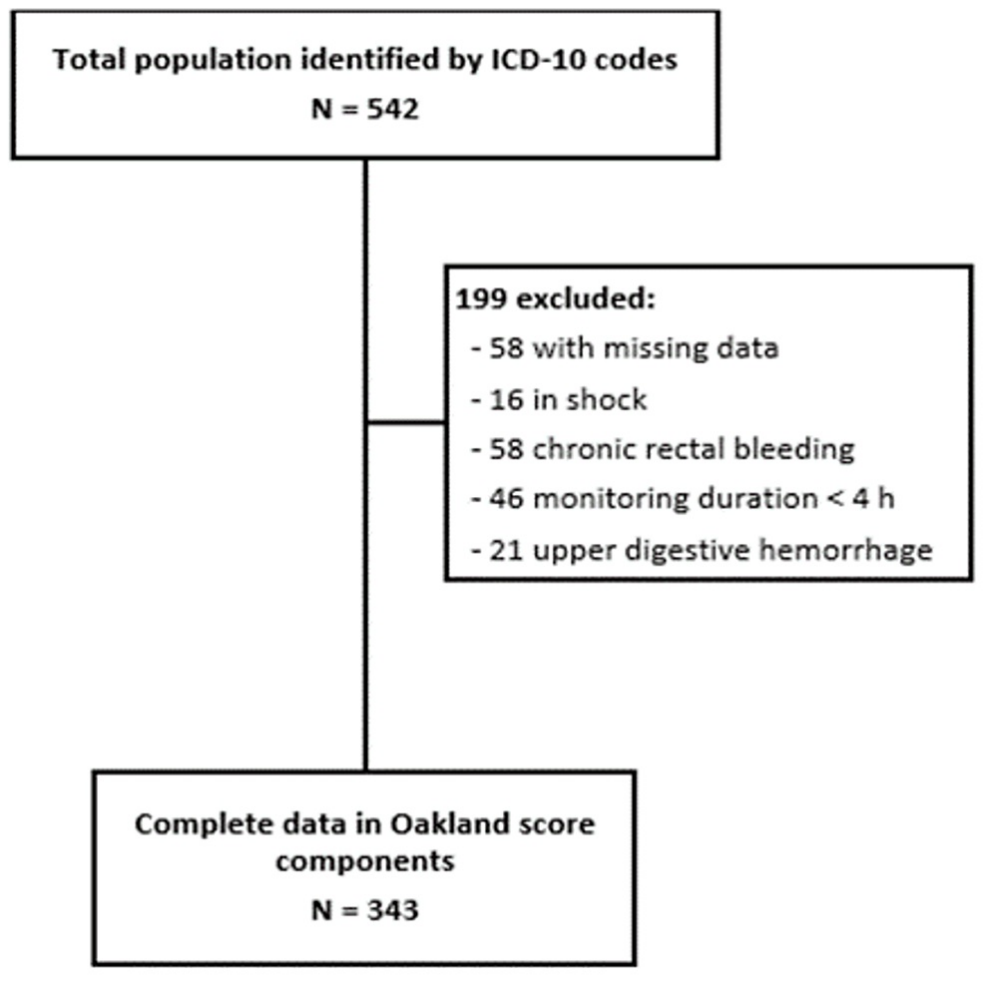
Flowchart of participant inclusion and exclusion

During the period from January 2, 2020, to August 30, 2023, 542 patients were admitted for hematochezia, but 199 patients were eliminated due to the presence of some exclusion criteria (Figure 1) leaving a study population of 343 cases (mean (SD) age: 68 (16.6); 152 women (44.3%)).

The majority of patients, 211 (61.5%; 95% CI, 56.1%-66.7%), experienced none of the adverse events described, making them eligible for safe discharge. A total of 108 patients (51.2%) in this group were men. Patients who satisfied the safe discharge criteria were younger (mean (SD) age, 76.8 (15.2) years vs. 63.6 (23.6) years, respectively) and had fewer prior hospital hospitalizations with LGIB (38 vs. 25 admissions) than patients who did not meet the safe discharge criteria (Table 1).

**Table 1:** Demographic characteristics and presenting features of patients admitted to the hospital LGIB: lower gastrointestinal bleeding

	General population (n=343)	Did not meet the criteria for safe discharge (n=132)	Met the criteria for safe discharge (n=211)	Correlation coefficient between the two sub-groups (p)
Age, mean (SD)	68.7 (21.7)	76 (15.2)	63.6 (23.6)	<0.001
Sex				
Male (%)	191 (55.7)	83 (62.9)	108 (51.2)	0.047
Female (%)	152 (44.3)	49 (37.1)	103 (48.8)	0.047
Previous hospital admission with LGIB (%)	63 (18.4)	38 (28)	25 (11)	<0.001
Hospitalized (%)	164 (47.8)	103 (78)	61 (28.9)	<0.001

**Figure 2:**
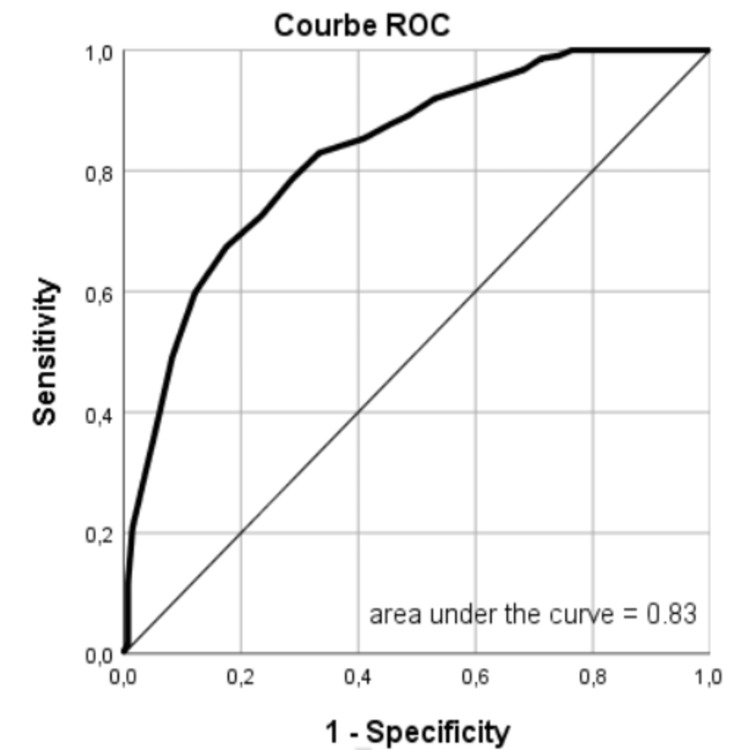
ROC curve for safe discharge ROC: receiver operating characteristic

The area under the receiver operating characteristic (AUROC) curve for the combined outcome of safe discharge was 0.83 (95% CI, 0.78-0.87), indicating strong discriminatory performance (Figure 2). In the predictive models for adverse events, the AUROC curves were as follows: for red blood cell transfusion, 0.88 (95% CI, 0.84-0.92), and for in-hospital rebleeding, 0.63 (95% CI, 0.56-0.70). The AUROC curve was not calculated for intra-hospital death, readmission within 28 days, and the requirement for therapeutic intervention due to the limited number of patients.

**Figure 3:**
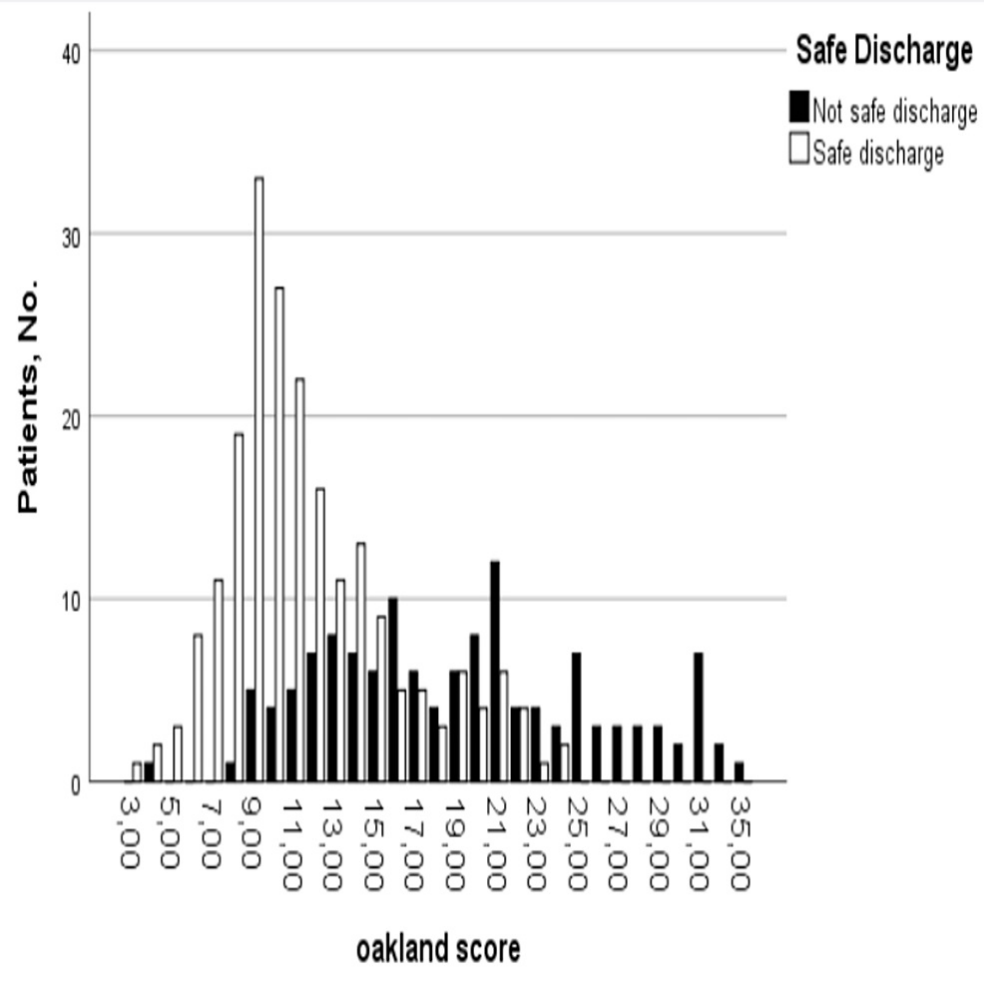
Total number of patients meeting the criteria for safe discharge by total Oakland score

The median Oakland score was 14 points (range, 3-35 points) (Figure 3). In total, 46 of 343 patients (13.4%) scored 8 points or lower (Table 2), with a specificity of 98.5% and a sensitivity of 20.9% for safe discharge (Table 3). A specificity of 95% for safe discharge was maintained to a score threshold of 9 points or lower, with a sensitivity of 36.5%. A total of 84 patients (24.5%) had a score of 9 points or lower.

**Table 2:** Comparative table of the different criteria of the Oakland score (retrospective criteria) RBC: red blood cell

	General population (n=343)	Patients with Oakland score ≤8 (n=46)	Patients with Oakland score ≤9 (n=84)
Rebleeding (%)	62 (18)	2 (4.4)	7 (8.3)
RBC transfusion (%)	77 (22.4)	0 (0)	1 (1.1)
Therapeutic intervention (%)	22 (6.4)	0 (0)	1 (1.1)
In-hospital death (%)	4 (1.1)	0 (0)	0 (0)
Readmission (%)	12 (3.4)	0 (0)	0 (0)
Patients meeting the criteria for safe discharge (%)	211 (61.5)	44 (95.7)	77 (91.7)
Patients not meeting the criteria for safe discharge (%)	132 (38.5)	2 (4.3)	7 (8.3)

**Table 3:** Sensitivity and specificity for safe discharge at different cutoff points CI: confidence interval; PPV: positive predictive value; NPV: negative predictive value

Cutoff	Patients n°	Sensitivity	95% CI	Specificity	95% CI	PPV	95% CI	NPV	95% CI
<2	0	0.00	0.0-1.7	100.00	97.2-100.0			38.5	38.5-38.5
≤3	1	0.47	0.01-2.6	100.00	97.2-100.0	100.0		38.6	38.4-38.8
≤4	4	1.42	0.3-4.1	99.24	95.9-100.0	75.0	24.0-96.6	38.6	38.1-39.2
≤5	7	2.84	1.1-6.1	99.24	95.9-100.0	85.7	42.2-98.0	39.0	38.3-39.6
≤6	15	6.64	3.7-10.9	99.24	95.9-100.0	93.3	65.1-99.1	39.9	39.0-40.9
≤7	26	11.85	7.8-17.0	99.24	95.9-100.0	96.2	77.4-99.5	41.3	40.1-42.6
≤8	46	20.85	15.6-27.0	98.48	94.6-99.8	95.7	84.4-98.9	43.8	42.0-45.6
≤9	84	36.49	30.0-43.4	94.70	89.4-97.8	91.7	84.0-95.9	48.3	45.5-51.0
≤10	115	49.29	42.4-56.2	91.67	85.6-95.8	90.4	84.1-94.4	53.1	49.5-56.6
≤11	142	59.72	52.8-66.4	87.88	81.1-92.9	88.7	83.1-92.7	57.7	53.4-61.9
≤12	165	67.30	60.5-73.6	82.58	75.0-88.6	86.1	80.8-90.1	61.2	56.2-66.1

The most common adverse outcomes in patients with Oakland scores of 9 points or lower were rebleeding (8.3%), transfusion, and endoscopic hemostasis in a single patient (1.2%), respectively. No patient died in this group and no patient was readmitted within 28 days (Table 4).

**Table 4:** Characteristics and outcomes of the false-positive cases according to an Oakland score ≤9

Characteristics	Proportion
Age, median (SD)	73.29 (20.5)
Hypertension (n)	6/7
Anticoagulation (n)	4/7
Rebleeding (n)	7/7
Transfusion (n)	1/7
Therapeutic intervention (n)	1/7
Readmission (n)	0/7
Hospitalization (n)	4/7
Deglobulization during the 24 hours (n)	0/7
Deaths (n)	0/7

## Discussion

The identification of very-low-risk patients who can be safely discharged early for outpatient management has become more important in recent times due to the growing demand for healthcare systems throughout the world. The decision to follow the patient on an outpatient basis can sometimes be difficult; therefore, the use of risk scores for the identification of this population seems relevant. From a patient safety perspective, the high specificity of those scores is of much greater importance than high sensitivity. Among the scores developed in this sense, the Oakland score appears to be the best at predicting safe discharge [9-13].

To our knowledge, this is the first study validating this score in a European population after the first validation study carried out in the United Kingdom [9]. A major point in our study is that only hematochezia was included as the primary symptom because bleeding from the right side of the colon was rarely associated with melena and that is more common in upper gastrointestinal bleeding or bleeding from the small intestine. 

We defined rebleeding as bleeding in the first four hours of evaluation; this definition was significantly correlated to the definition used in previous validation studies that define rebleeding as additional blood transfusions or a decrease in hematocrit concentration of 20% or more after 24 hours of clinical stability [14], and we used the first definition primarily because choosing to treat patients who have been admitted more than 24 hours runs the risk of limiting our selection to the most seriously ill patients.

In Table 1, we note 22% of inappropriate returns as well as 28.9% of excess hospitalizations which underlines the interest in using a prognostic score complementary to clinical judgment. We also note the male predominance and high age as risk factors for LGIB requiring hospitalization, as in the derivation series [9] of the Oakland score.

Our findings demonstrate like previous validation studies [6-9] that the Oakland score exhibited discriminatory capability, enabling the identification of patients at a low risk of encountering adverse outcomes, thus indicating their suitability for hospital discharge. Additionally, we observed that the previously suggested threshold of 8 points or lower effectively identified 13% of patients appropriate for outpatient follow-up, demonstrating good specificity. This number of positive tests appears low and therefore calls into question the usefulness of this threshold. However, the cutoff could be extended to 9 points or lower, making it possible to detect a higher proportion of patients (24.4%) safe to be discharged and to increase the sensitivity to 36% while maintaining an accepted specificity (95%). These results are consistent with those of a 2022 meta-analysis [13] which included the prospective derivation study conducted in the United Kingdom [9] and a large-scale study in the United States [15]. Overall, the score seems more useful at the threshold of 9 but with a global risk of error in 8% of patients. 

Therefore, patients with LGIB who receive an Oakland score of 9 or lower can be safely discharged for outpatient management with a considerable level of confidence that they will not experience adverse outcomes while receiving ambulatory care. The good specificity was attained with a corresponding decrease in sensitivity, which is acceptable from a clinical perspective. Indeed, a very specific risk score would reduce the number of false-positive cases, which consist of patients who were initially considered safe for discharge but later experienced a negative outcome or an unsafe discharge. In contrast, low sensitivity would lead to more false-negative cases or patients who were first thought to be unsuitable for discharge but eventually did not have a negative outcome. The former should take precedence over the latter when weighing the need to reduce unsafe discharges and unnecessary hospitalizations.

We discussed the seven false-positive cases that were found to further evaluate the test's reliability at this threshold of 9 (Table 4). A correlation study was not possible because of the small number of cases: Rebleeding within the first four hours of admission was noted in all seven cases, but it wasn't associated with deglobulization, despite being identified as an independent risk factor for severe LGIB [14]. Furthermore, none of the seven patients died or needed readmission, which may suggest that rebleeding was not significant in those cases. Finally, because all of the patients had high blood pressure, this may also have contributed to their lower Oakland scores.

If the false-positive rate increases rapidly with the threshold level, it is undoubtedly because certain prognostic factors are not identified by previous studies. Prognostic factors for LGIB remain a topic of prospective work at this stage.

Among the possible limitations, note that this is a retrospective study; therefore, it's likely that patients who were discharged later received medical care in private hospitals and that data were not included in the analysis. We also note a few complications in the population with a threshold lower than 9, so there is a risk of overestimation of the specificity.

## Conclusions

The Oakland score can be used to determine which LGIB patients have a low probability of adverse outcomes and can, thus, be safely discharged from the emergency room. To identify a higher percentage of low-risk patients while maintaining sensitivity, the initial Oakland score threshold of 8 points can be increased to 9 points; the selection of this threshold should depend on the validation from other large series, specifically confirming that false positives at this threshold do not result in a loss of opportunity for patients.
